# Next-generation sequencing yields a complete mitochondrial genome of the Tartar sand boa (*Eryx tataricus*) from Junggar Basin

**DOI:** 10.1080/23802359.2020.1715279

**Published:** 2020-01-20

**Authors:** Bo Cai, Xianguang Guo, Zhaobin Song, Jianping Jiang

**Affiliations:** a Chengdu Institute of Biology, Chinese Academy of Sciences, Chengdu, China;; b College of Life Sciences, Sichuan University, Chengdu, China;; c University of Chinese Academy of Sciences, Beijing, China;; d Key Laboratory of Bio-resources and Eco-environment of Ministry of Education, College of Life Sciences, Sichuan University, Chengdu, China

**Keywords:** *Eryx tataricus*, Henophidia, mitochondrial genome, next-generation sequencing, phylogenetic tree

## Abstract

The viviparous Tartar sand boa, *Eryx tataricus*, is a widespread species in arid Central Asia. A complete mitochondrial genome of one individual from Junggar Basin in Northwest China was determined by next-generation sequencing. The mitogenome is 17,537 bp in size, comprising 2 ribosomal RNA genes, 13 protein-coding genes (PCGs), 22 transfer RNA genes (tRNAs), and 2 control regions. The order and structure of the genes are similar to those of other Henophidia snakes. Phylogenetic analysis based on 13 concatenated PCGs recovered the monophyly of Boidae and indicated that *E. tataricus* is closely related to *Boa constrictor* plus *Eunectes notaeus*.

The viviparous Tartar sand boa, *Eryx tataricus*, is a widespread species in arid Central Asia, including the five post-Soviet Central Asian countries, northern Iran and Afghanistan, Northwest China, and southern Mongolia (Ananjeva et al. 2006). The advancement of sequencing technology including the next-generation sequencing (NGS) has facilitated the rapid obtainment of mitochondrial genome from various animals (Hahn et al. 2013). In this study, we determined a complete mitochondrial genome of *E. tataricus* using NGS reads through Illumina HiSeq 2000 platform.

The snake was collected from Junggar Basin (N44.54°, E82.58°) in July 2009, which is the second-largest inland basin in China. The Basin is situated in northern Xinjiang and bounded by the Altay Mountains to the northeast and the Tianshan Mountains to the south. The specimen (field number Guo563) was deposited in the herpetological collection, Chengdu Institute of Biology, Chinese Academy of Sciences. Genomic DNA was extracted from liver tissue using Trelief Animal Genomic DNA Kit (Tsingke, Beijing, China) using the protocol prescribed by the manufacturer. Then, the genomic DNA was shipped to Tsingke (Chengdu, China) for PE150 library construction and sequencing on an Illumina HiSeq 2000 instrument. *De novo* assembly of clean reads was performed using SPAdes v3.11.0 (Bankevich et al. 2012). Then, we took a similar strategy as described previously (Chen et al. 2019) to assemble and annotate the complete mitogenome of *E. tataricus*. It was deposited in GenBank with accession number MN646174.

The complete mitogenome consisted of 17,537 bp and contained 13 protein-coding genes (PCGs), 22 tRNA genes, two rRNA genes, and two control regions (CR or D-loop). The gene arrangement and composition was identical with the published mitogenome of *E. tataricus* (Hu et al. 2019), exhibiting a typical snake mitochondrial genome feature (Dong and Kumazawa 2005; Yan et al. 2008). Most genes were encoded by the H-strand except for *ND6* and eight tRNA genes (*tRNA-Gln, Ala, Asn, Cys, Tyr, Ser^UCN^, Glu,* and *Pro*). Six of the PCGs were initiated with the typical ATG codon, except for *ND1* with ATA, *ND2* and *ND3* with ATT, *COX1*, *COX2*, *ATP6*, and *ND5* with GTG. Meanwhile, most PCGs were terminated with the typical TAA/TAG/AGA/AGG codon, except for *ND1, COX3*, *ND3* and *CYTB* with the incomplete termination codon T. The two CRs (1,076 bp and 1,214 bp in length) were located between *tRNA-Pro* and *tRNA-Phe* for CR1 and between *tRNA-Ile* and *tRNA-Leu^UUR^* for CR2, respectively.

The concatenated PCGs of Heophidia available in GenBank and one Colubridae taxon as outgroup were used to reconstruct the Bayesian phylogenetic tree for assessing mitochondrial sequence authenticity of *E. tataricus* and its phylogenetic placement. The phylogenetic tree recovered the monophyly of Boidae (Figure 1). The two individuals of *E. tataricus* clustered together and were closely related to *Boa constrictor* plus *Eunectes notaeus*. The overall phylogenetic relationships among Henophidia was in line with previous studies (Douglas and Gower 2010; Reynolds et al. 2014). The complete mitogenome of *E. tataricus* will provide fundamental data to explore the mitochondrial genome evolution in sand boas.

**Figure 1.**
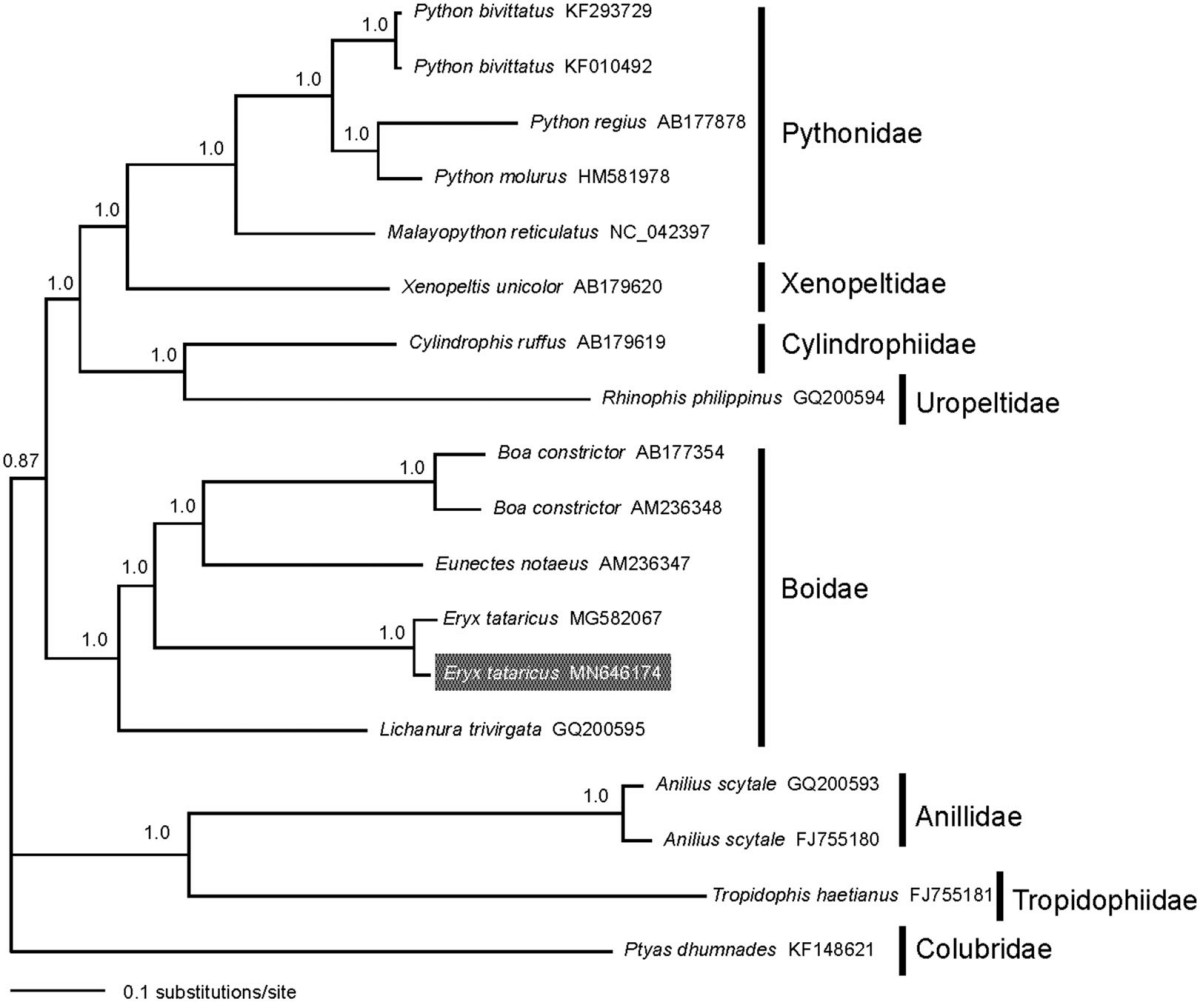
A majority-rule consensus tree inferred from Bayesian inference using MrBayes v.3.2.2 (Ronquist et al. 2012) under the GTR + G substitution model, based on the concatenated PCGs of 17 snakes of Heophidia and one outgroup of Colubridae. The newly sequenced sample was highlighted in gray. DNA sequences were aligned in MEGA v.6.06 (Tamura et al. 2013). The PCGs were translated into amino acids sequences, and were manually concatenated into a single nucleotide dataset (in total 11,365 bp). Node numbers show Bayesian posterior probabilities. Branch lengths represent means of the posterior distribution. GenBank accession numbers are given with species names.
